# Determination of Mechanical and Tribological Properties of Silicone-Based Composites Filled with Manganese Waste

**DOI:** 10.3390/ma14164459

**Published:** 2021-08-09

**Authors:** Maciej Mrówka, Anna Woźniak, Jerzy Nowak, Gabriel Wróbel, Sebastian Sławski

**Affiliations:** 1 Department of Theoretical and Applied Mechanics, Silesian University of Technology, Konarskiego 18A, 44-100 Gliwice, Poland; maciej.mrowka@polsl.pl (M.M.); gabriel.wrobel@polsl.pl (G.W.); 2 Biotechnology Center, Silesian University of Technology, Krzywoustego 8, 44-100 Gliwice, Poland; 3 Department of Engineering Materials and Biomaterials, Silesian University of Technology, Konarskiego 18A, 44-100 Gliwice, Poland; anna.wozniak@polsl.pl; 4 Zakłady Górniczo-Hutnicze “Bolesław” S.A. Capital Group, Kolejowa 37, 32-332 Bukowno, Poland; jnowak@mail.zghboleslaw.pl

**Keywords:** silicone, composite, casting, manganese residue, mechanical properties, abrasion, waste management

## Abstract

High-tonnage industrial processes generate high amount of waste. This is a growing problem in the whole world. Neutralizing such waste can be time consuming and costly. One of the possibilities of their reuse is to use them as fillers in polymer composites. Introduction of the filler in polymer matrix causes change in its mechanical and tribological properties. In the article, the effect of introducing fillers from post-production waste, and its effect on changing the physical properties of silicone-based composites filled with manganese (II) oxide and waste manganese residue was investigated. The composites were made by gravity casting. Composites with 2.5, 5, 7.5, and 10 wt% of the fillers were examined. The composite materials were subjected to tests such as: density, hardness, resilience, tensile test, abrasion resistance, and ball-on-disc. Microscopic images showed that, the particles of the fillers are uniformly distributed in silicone matrix with the formation of smaller agglomerates. Such agglomerates introduced a discontinuity in the structure of the polymer material, which caused a decrease in the tensile strength and elongation at break for all tested compositions in comparison with the mechanical properties of the silicone used as the matrix. However, it was found that all silicone-based composites filled with manganese (II) oxide and manganese residue showed a reduction in abrasive wear, compared to the reference sample.

## 1. Introduction

The growing amount of production waste has caused scientists to investigate new ways of recycling [1]. Many inorganic compounds are found in industrial waste that have been used as fillers in composites, whose matrices are polymers [2,3]. They could be used in some modern applications [4,5,6,7]. Compounds that use these fillers demonstrate changed physicochemical properties in relation to the base material [4,5,6,7]. Research on these composites investigates whether the addition of inorganic compounds can reduce wear, compared to the material used as the matrix. The literature describes the effect of introducing Al_2_O_3_, TiO_2_, ZnO, CuO, SiC, ZrO_2_, Si_3_N_4_, SiO_2_, B_6_O_18_Zn, Mg_3_(OH)_2_Si_4_O_10_, and CaCO_3_ particulate fillers, which have been incorporated into PPS (polyphenylene sulfide), PEEK (polyether ether ketone), epoxy, PMMA (polymethyl methacrylate), and PTFE (polytetrafluoroethylene) matrices, to improve their wear performance [8,9,10,11,12,13,14]. Other tests show that the combination of the additives, Al_2_O_3_ and CaSiO_3_, with epoxy improved its wear resistance [15]. In most cases, the optimum in-organic particle filler could be identified by testing which of these produced the highest wear resistance. Another important aspect when investigating new materials is their toxicity to the environment, this includes both the materials themselves and their degradation products [16,17].

There are several examples of silicone-based composites in the literature. Most of the publications concerning composites with silicone matrix are from the last 5 years. So, interest in this type of materials increased in recent years. Various additives have been introduced to silicone to obtain various types of physicochemical, mechanical, and biological improvements, depending on the intended use of a given composite material.

In studies from 2011, the authors obtained composites that were used as cable sheaths, owing to the addition of quartz and wollastonite to silicone [18]. In other studies, the addition of nickel to the silicone matrix improved the mechanical properties of the composites, compared to when the silicone alone was used as the matrix [19].

Research by Masłowski et al. showed that the addition of magnetite and ionic liquids to silicone resulted in the composites having increased tensile strength, elongation at break, and stability during thermal and photo oxidative aging [20,21]. The studies of Imiela et al. have shown that ceramic-containing silicone composites age well, and are therefore suitable for use as cable sheaths [22]. Fan et al. on the other hand, studied phosphorus-silicone composites used in white light-emitting diodes. The composites were shown to be resistant to the aging process in moist conditions, and have a higher Young’s modulus, compared to pure silicone [23].

Jin et al. showed that the addition of graphene oxide protects silicone composites against fouling from sea plants, while also decreasing the Young’s modulus of the new composite [24]. Beter et al. used glass fibers as reinforcement with a silicone matrix. The addition of glass fibers resulted in a 30 times higher stiffness and a 10 times higher breaking stress [25]. Song et al. studied composites filled with different contents of ZrSi2. The results showed that the vulcanization time of silicone rubber may be reduced by introduction of ZrSi_2_. With the increase in the percentage of ZrSi_2_, the tensile strength first increased and then decreased [26].

Mrówka et al. researched silicone-based composites filled with wood waste. The obtained materials were characterized by reduced abrasion and may be useful in seabed conditions [27].

This study is a continuation of previous research [28] in which authors investigated the introduction of zinc waste as a filler in silicon-based composites and its effect on the mechanical and tribological properties of the material. The obtained results showed that for 5 to 20% filling, the addition of both the zinc dust, and the sifted and non-sifted zinc ash improved the tribological properties and reduced the abrasive wear of the material.

Encouraged by these results, the authors obtained manganese oxide and manganese residue from the Zakłady Górniczo-Hutnicze “Bolesław” S.A. Capital Group (Bukowno, Poland). These materials are waste products of the electrolysis process, when used with steel-lead alloy anodes. The authors examined the benefit of these waste products as fillers on the mechanical and tribological properties of composites, whose matrix was the same silicone that was used in the previous research. The use of waste materials from high-volume industrial processes is an important part of caring for the natural environment. Currently, paths for the recycling of waste in various branches of the economy are being sought. This will reduce the need to store and process industrial waste. The usage of waste materials from manganese processing as fillers is not mentioned in the available literature. The authors expect that, when used as a filler, the manganese residue (MR) product that is left over from the electrolysis reaction process, with anodes made of a silver-lead alloy, will reduce the abrasion of silicone-based composites. The physicochemical properties of the manufactured composites will be assessed, when both manganese (II) oxide and manganese residue are used as a filler.

## 2. Materials and Methods

### 2.1. Materials

Feingosil 128 PU [29] was used as a matrix. It is condensation-cured RTV-2 silicone rubber. The properties of used matrix are presented in Table A1 (Appendix A). Manganese(II) oxide (MnO) was obtained from the company Oxymine S.A. Oxydes Minéraux de Poissy (Poissy, France). The sieve analysis showed: 100% of the grain size < 0.1 mm. Chemical analysis of Manganese(II) oxide type K.M.: 78% MnO, 5.9% Fe, 2.7% CaO, 1.5% Al_2_O_3,_ 1.3% MnO_2_, and 10.6% the rest substances. Manganese residue as a product of the electrolysis reaction process on anodes made of a silver-lead alloy was provided by Zakłady Górniczo-Hutnicze “Bolesław” S.A. Capital Group (Bukowno, Poland). The sieve analysis showed: 92.5% of grains < 0.1 mm and 7.5% of grains 0.1–0.315 mm. Chemical composition of the manganese residue provided by the chemical laboratory of Zakłady Górniczo-Hutnicze “Bolesław” shows: 72% MnO_2_, 11% SiO_2_, 7% MnO, 5.5% ZnSO_4_, and 4.5% the rest substances. 

### 2.2. Composites Preparation

**Figure 1:**
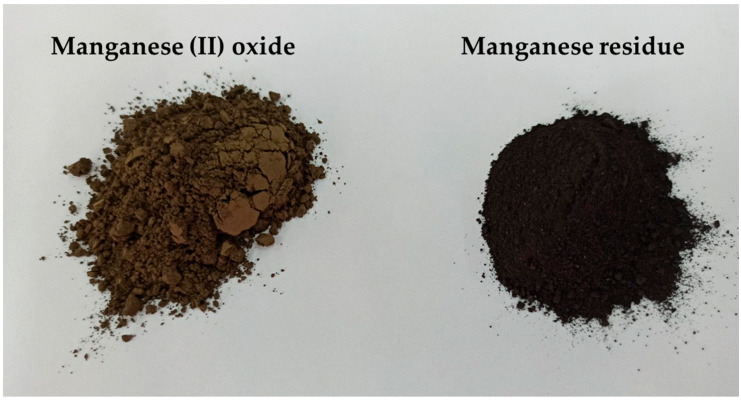
Materials used as fillers.

In case of the research concerning the impact of the zinc waste fillers on change in tribological and mechanical properties of silicone-based composites [28] authors examined the compositions with 5 wt%, 10 wt%, and 20 wt% of the fillers. Provided research showed that, the composites with 20 wt% of the fillers have worst mechanical and tribological properties in comparison with the composites with 5 wt% and 10% of the same filler. The best results were obtained for the composites with 5 wt% of the fillers. Based on these results authors decide to examine the composites with 2.5, 5, 7.5, and10 wt% of the fillers. Gravity casting method was used to prepare silicone-based composites. The fillers were gradually heat-treated at 110 °C for 3 h, before adding into silicone matrix, until a constant weight was obtained. Materials that are used in the research as fillers are presented in Figure 1.

The compositions of silicone component A, fillers and catalyst were mixed using a high-shear mixer with speed of 500 RPM. The mixing time of the compositions was 5 min. Amount of the introduced ingredients of each composition was precisely weighted using BTA2100D precise scale (AXIS Sp. z.o.o., Gdańsk, Poland) with 0.01 g accuracy. Prepared compositions were poured into wooden molds. After 72 h the samples were cut by punching. The compositions and their designations are shown in Table 1. 

**Figure 2:**
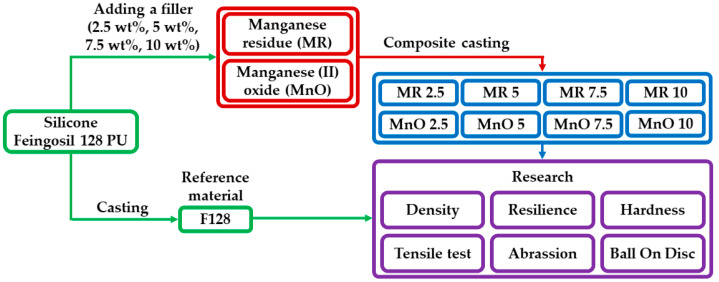
Scheme of research methodology.

The obtained samples were subjected to mechanical tests. Densities were measured using hydrostatic weighing tests. Additional characterizations, abrasion resistance tests (according to Schopper-Schlobach method), ball-on-disc tests, hardness tests (Shore type A), and tensile tests were carried out. In case of density, hardness, and tensile test five samples from each composition for each test were prepared. In case of resilience, abrasion resistance and ball-on-disc test three samples from each composition for each test were prepared. Figure 2 shows the scheme of developed research methodology. All tests were conducted at 22 °C temperature and 50% humidity. The results of all the tests for silicone F128 were taken from the previous publication [28].

### 2.3. Research Methods

#### 2.3.1. Density Testing 

Densities of prepared composites were determined by hydrostatic weighing. Test was conducted in accordance with EN ISO 1183-1:2006 [30] using five samples from each composition. Samples were weighed using an Adventure Pro AV264CM (OHAUS Europe GmbH, Nänikon, Switzerland) analytical balance with a density measurement kit. Each sample was weighed twice. The first measurement was carried out with a sample placed on a pan in the air. The second measurement was carried out for a sample that was immersed in water (0.997 g/cm^3^). The densities of the composites were determined using Equation (1):

(1) $$\rho =\rho_{H_{2}O}\times \frac{m_{1}}{\left(m_{1}-m_{2}\right)},$$

where:

- $\rho_{H_{2}O}$—density of water (g/cm^3^);
- $m_{1}$—dry sample mass (g);
- $m_{2}$—wet sample mass (g).

#### 2.3.2. Rebound Resilience 

The resilience of the samples was measured by Schober’s test. Resilience was determined in accordance with the EN ISO 4662:2017 [31] standard. Test was performed for three samples with dimensions of 30 mm × 30 mm × 5 mm on a VEB Rauenstein EPGi (WPM Veb Thuringer Industriewerk, Rauenstein, Germany) apparatus [31]. The measurement of resilience using this method relies on reading the value indicated by the pointer after hitting into tested sample.

#### 2.3.3. Hardness Test

The hardness of prepared composites was measured in Shore A scale. Test was performed in accordance with EN ISO 7619-1:2010 [32] standard. Shore A type Zorn (Zorn Instruments GmbH & Co., Hansestadt, Germany) hardness durometer was used to perform hardness measurements. Each composite was measured five times. During the test, a distance of at least 10 mm from the sample edge was maintained.

#### 2.3.4. Tensile Test

A tensile test was performed in accordance with EN ISO 527-1 [33] standard. The measurements were made for five samples (type 5-B) from each of the prepared composite. Instron 4465 (Instron, Norwood, MA, USA) testing machine was used during the test. The test was performed with a speed of 500 mm/min. Based on the obtained results tensile strength and elongation at break for all prepared compositions were determined.

#### 2.3.5. Abrasion Resistance Tests

The abrasion resistance test was performed in accordance with the Schopper-Schlobach method. Test was provided in accordance with the EN ISO 4649:2007 [34] standard on an APG Schopper-Schlobach apparatus (APG Germany GmbH, Friedberg, Germany). During the research, sandpaper with grit of 60 was used. Sandpaper was wound on a roller with a diameter of 150 mm. Rotational speed of the roller was 40 RPM. The abrasion resistance (abrasive wear), i.e., the volume loss relative to a standard sample, was determined for three cylindrical samples cut out from each of the prepared composites. Abrasive wear resistance was calculated as the volume loss of the abraded sample based on Equation (2):

(2) $$\Delta V=\frac{m_{1}-m_{2}}{\rho },$$

where:

- ρ—sample density (g/cm^3^);
- $m_{1}$—mass of sample before abrasion (g);
- $m_{2}$—mass of sample after abrasion (g).

#### 2.3.6. Ball-on-Disc

A friction test was conducted using the CSM tribometer (CSM Instruments, Needham, MA, USA) using the ball-on-disc method. A stainless steel ball with a diameter of 6 mm was used as a counter specimen. The friction test was carried out using the normal load F_n_ of 0.5 N for 1 Hz frequency. The measured distance was 20 m. Based on the performed measurements, the value of the coefficient of friction (µ) was determined.

## 3. Results

**Figure 3:**
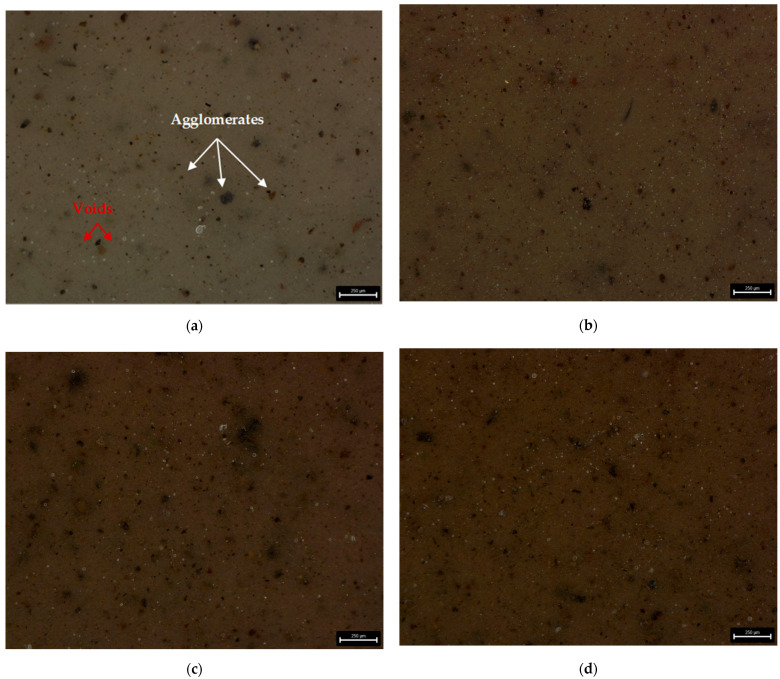
Microscopic images of silicone-based composites with MnO filler content: (**a**) 2.5 wt%; (**b**) 5 wt%; (**c**) 7.5 wt%; (**d**) 10 wt%.

**Figure 4:**
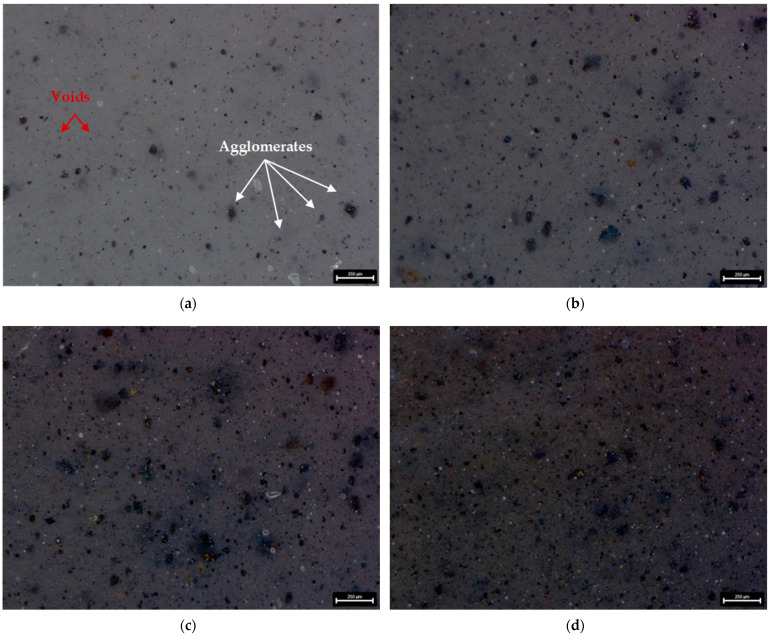
Microscopic images of silicone-based composites with MR filler content: (**a**) 2.5 wt%; (**b**) 5 wt%; (**c**) 7.5 wt%; (**d**) 10 wt%.

**Figure 5:**
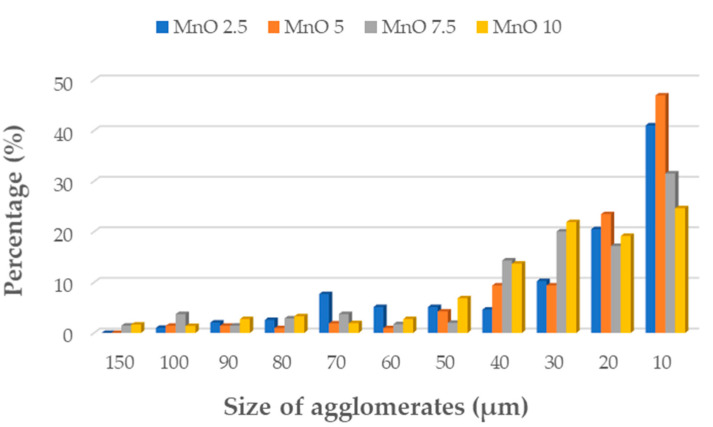
Size of agglomerates in silicone-based composites filled with MnO.

**Figure 6:**
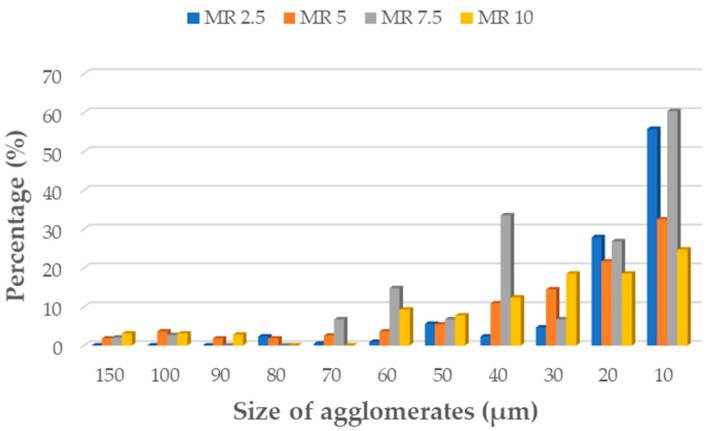
Size of agglomerates in silicone-based composites filled with MR.

For surface topography and morphology examination the digital 3D microscope DVM6 (Leica Micorsystem, Wetzlar, Germany) was used. As seen from digital microscopic graphs, particles of the manganese oxide (MnO series—Figure 3) and manganese residue (MR series—Figure 4) are uniformly distributed in silicone matrix with the formation of smaller agglomerates. Of course, based on the obtained results it can be seen that with increasing concentration of filler, the number of agglomerates increased too. The number of agglomerates was counted and their parameters, i.e., length were measured for each series of the prepared composites. Area fraction (Af) measurement function was used to evaluate the size of agglomerates distribution in tested samples. Based on the agglomerates size measurements, it can be seen that for both MnO (Figure 5) and MR (Figure 6) samples series, with increase concentration of filler, the MnO- and MR particles tend to form larger agglomerates in the matrix. For samples, with 5 and 7.5 wt% of MnO-filled silicone composites, the agglomerates with length more than 150 µm were observed. Similar trend was observed for MR-filled silicone composites. However, for both fillers, the largest share in the volume of agglomerates with a size in the range of 40–10 µm was observed. For samples with 10 wt% concentration of filler, smaller agglomerates were visible. Additionally, for all tested samples some voids also can be seen in the composites. The images were taken at magnification ×150.

### 3.1. Density Testing by Hydrostatic Weighing

**Figure 7:**
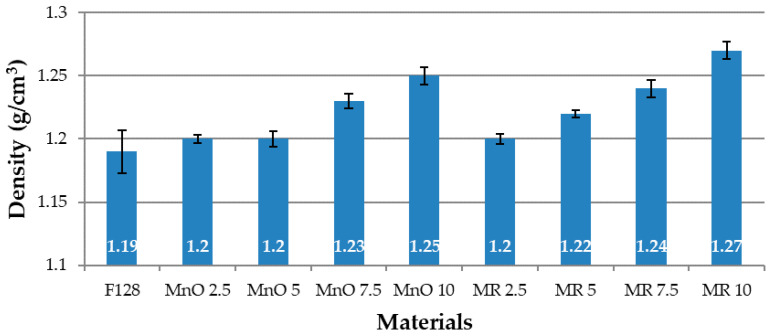
Densities of tested materials.

Figure 7 shows the results of the density test of the obtained composites and of the silicone F128, which was used as a matrix.

For all the composite materials, an increase in density was noted and as compared to the samples made of native silicone (1.19 g/cm^3^). For the MnO 2.5 and MnO 5 materials, the density was slightly higher than that for the base material and in both materials it was 1.2 g/cm^3^. The MnO 7.5 material had a density of 1.23 g/cm^3^, while the MnO 10 material had a density of 1.25 g/cm^3^. For the materials in which the filler was manganese residue, it was also noticed that, as the filler content in the composite increased, the material density also increased. The MR 2.5 material had a density equal to the MnO 2.5 material (1.2 g/cm^3^). The MR 5 material had a density of 1.22 g/cm^3^, while MR 7.5 had a density of 1.24 g/cm^3^. The MR 10 material was characterized by the highest density among the obtained composites (1.27 g/cm^3^). The increase in the density of composite materials is related to the fact that the density of the introduced filler is greater than that of silicone. The greater the weight fraction of the filler, the greater the increase in material density.

### 3.2. Rebound Resilience 

**Figure 8:**
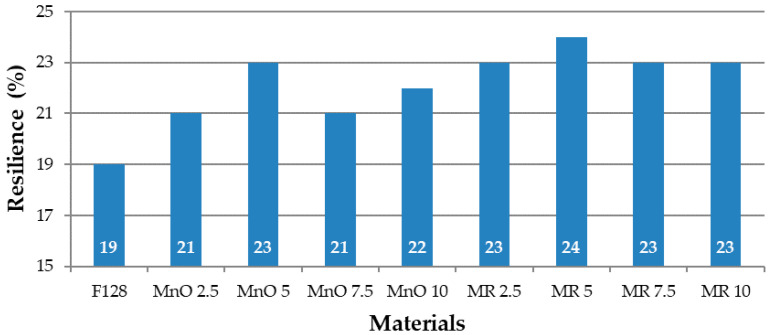
Resilience values of tested materials.

The values of resilience determined in accordance with Schober’s test for the tested compositions are presented in Figure 8.

The value of resilience for the base material F128 is 19%, the lowest value among all tested materials. For the MnO 2.5 and MnO 5 composites, an increase in the value of resilience was recorded—21 and 23%, respectively. The MnO 7.5 material showed a decrease in resilience of 21%, while the MnO 10 material showed a slight increase, in comparison to the MnO 7.5 material (22%). For composites in which the filler was manganese residue, for MR 2.5 material, the rebound value was 23%. For MR 5, there was an increase in the value of resilience to 24%. The MR 7.5 and MR 10 materials were characterized by a slightly lower but equal rebound, amounting to 23%. As such, composites containing a manganese residue filler have similar values of resilience. The composite resilience increase in comparison to pure silicone is related to the use of fillers. The stiffness in the case of fillers is lower than that for silicone. Thus, the silicone mixed with fillers is more resilient than neat silicone.

### 3.3. Hardness Test 

**Figure 9:**
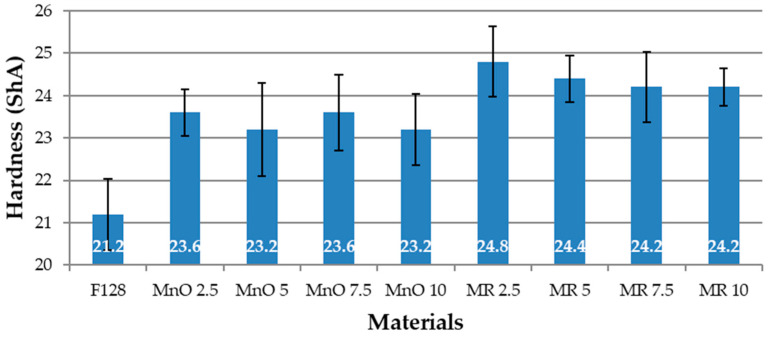
Hardness of tested materials.

The hardness values for the composite materials and the silicone matrix material are presented in Figure 9.

The hardness of the samples made of unmodified F128 silicon was 21.2 ShA. For the remaining composite samples, the hardness values were higher compared to F128. For the composites in which the filler was MnO, the hardness values were as follows: MnO 2.5—23.6 ShA, MnO 5—23.2 ShA, MnO 7.5—23.6 ShA, and MnO 10—23.2 ShA. These values are relatively similar. The filler content in the composite in the range of 2.5 to 10% provides the composite materials with a comparable hardness value. For composite materials in which the filler was manganese residue (MR), the hardness was also higher than that of F128 and amounted to MR 2.5—24.8 ShA, MR 5—24.4 ShA, MR 7.5—24.2 ShA, and MR 10—24.2 ShA. A slight decrease in hardness with increasing filler content was observed. Moreover, here it can be assumed that the hardness values for materials filled with manganese residue have a similar value. The increase in the hardness of composites along with the increase in the amount of filler can be explained by the hardness of the fillers. They are hard substances, and their addition increases the hardness of the composite in comparison to silicone F128.

### 3.4. Tensile Test

**Figure 10:**
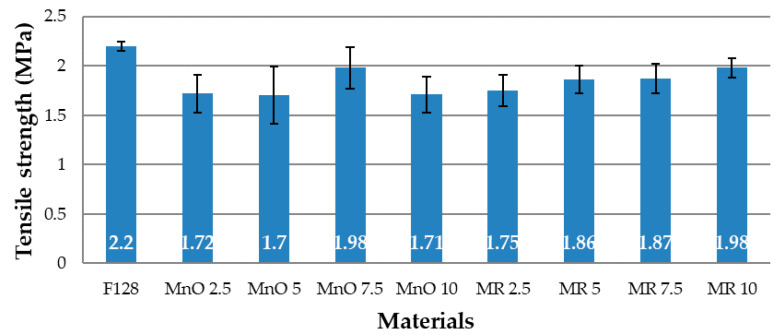
Tensile strength.

**Figure 11:**
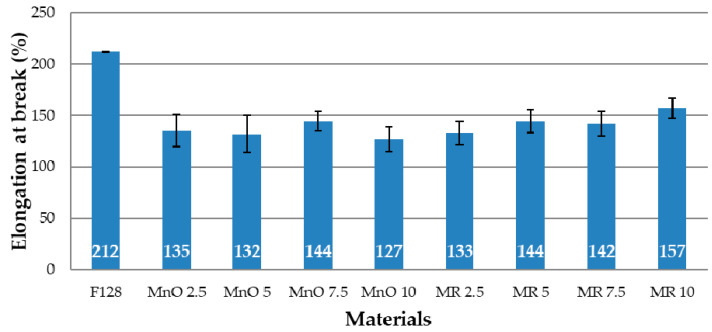
Elongation at break.

**Figure A1:**
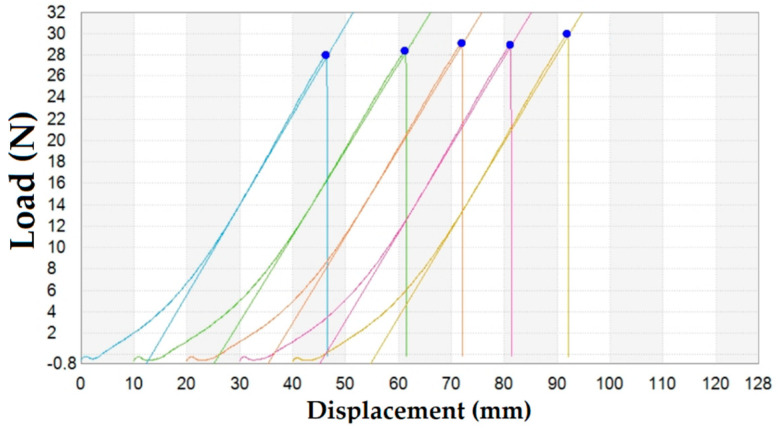
The load–deformation curves of the F128 material.

**Figure A2:**
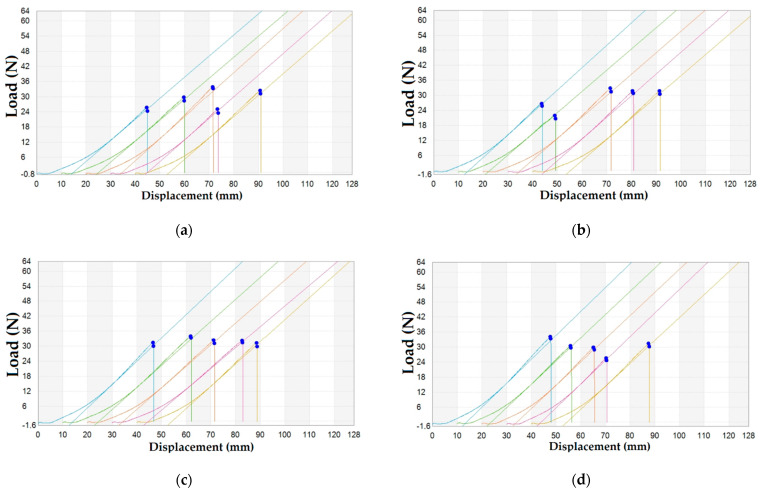
The load–deformation curves of the silicone-based composites with MnO filler content: (**a**) 2.5 wt%; (**b**) 5 wt%; (**c**) 7.5 wt%; (**d**) 10 wt%.

**Figure A3:**
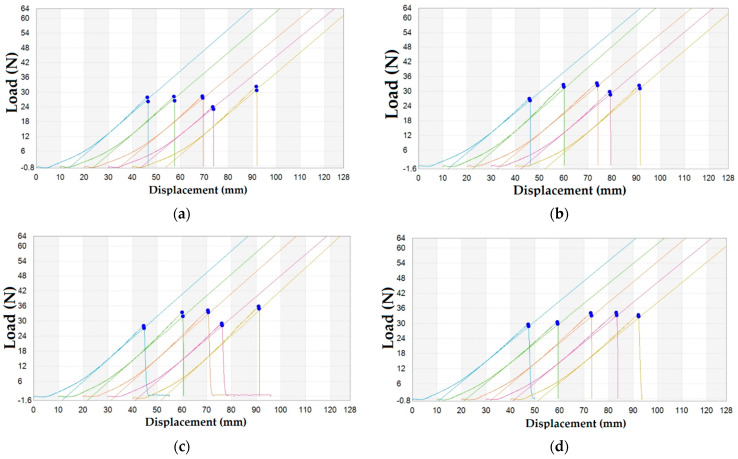
The load–deformation curves of the silicone-based composites with MR filler content: (**a**) 2.5 wt%; (**b**) 5 wt%; (**c**) 7.5 wt%; (**d**) 10 wt%.

The mechanical properties for all tested compositions are shown in Figure 10 and Figure 11. Figure 10 shows the tensile strength values, while Figure 11 shows the elongation at break values. The load–deformation curves of the pure silicone (F128) and silicone-based composites filled with MnO and MR are presented in Figure A1, Figure A2 and Figure A3 (Appendix B).

The tensile strength value for F128 silicone is 2.2 MPa, the highest value among all the tested materials. For the MnO 2.5 material, a decrease in the tensile strength value by 22% was observed, compared to the base material (1.72 MPa). The tensile strength value for the MnO 5 material, equal to 1.7 MPa, is similar to the value for MnO 2.5. For the MnO 7.5 material, a significant increase in the tensile strength value was observed in comparison with the two composites containing 2.5 and 5% of the filler in the structure. MnO 7.5 is characterized by a tensile strength value equal to 1.98 MPa. For the MnO 10 material, the tensile strength value decreased again to a value similar to that of MnO 2.5 and MnO 5 (1.71 MPa). For the composites that used a manganese residue filler, it was noticed that tensile strength increased, along with concentration of the filler. The tensile strength value for MR 2.5 is 1.75 MPa, MR 5—1.86 MPa, MR 7.5—1.87 MPa, and MR 10—1.98 MPa. The tensile strength value for MR 10 is equal to the MnO 7.5 value and both these values represent the highest tensile strength values among the tested materials.

The elongation at break value for F128 silicone has the highest value of all tested materials (212%). All composite materials are characterized by an elongation at break, which is lower than that of the silicone used as a matrix. For the MnO 2.5 material, the elongation at break value is 135%, and for MnO 5—132%. For the MnO 7.5 material, an increase in elongation at break to the level of 144% is observed, but for MnO 10 a decrease to the level of 127% is observed. The elongation at break value for the MnO 10 material is the lowest among all tested materials and is over 40% lower than the elongation at break value for F128. For materials where manganese residue was used as a filler, the MR 2.5 material has elongation at break equal to 133%, while MR 5 and MR 7.5 materials have similar values of 144 and 142%, respectively. For MR 10 material, an increase in the value of elongation at break to 157% is observed. The elongation at break value for MR 10 is the highest value among all composite materials produced. The decrease in the tensile strength and elongation at break for composites in comparison to the reference sample (F128) can be explained by the uneven distribution of filler grains in the matrix and their agglomeration. This may adversely affect the structure of the polymer chains, which lowers the mechanical properties. Microscopic images of the samples (Figure 3 and Figure 4) showed that the filler grains, especially at 5 and 7.5 wt% content, tend to agglomerate in the matrix. It introduces a discontinuity in the structure of the polymer material, which may translate into a decrease in the tensile strength and elongation at break for all composite materials in relation to the mechanical properties of the silicone used as the matrix.

### 3.5. Abrasion Resistance Tests

**Figure 12:**
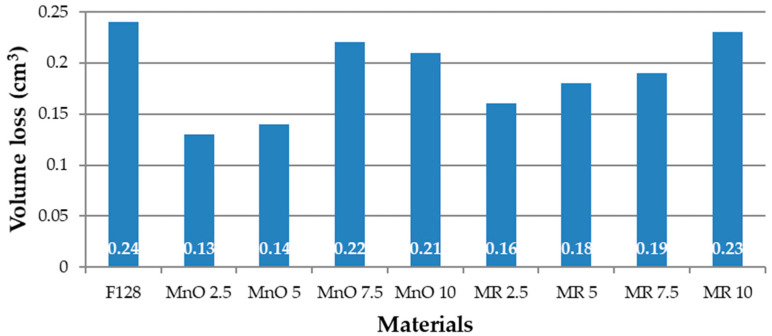
Volume loss of tested materials.

The results of the abrasive wear tests as a samples volume loss are presented in Figure 12.

**Figure 13:**
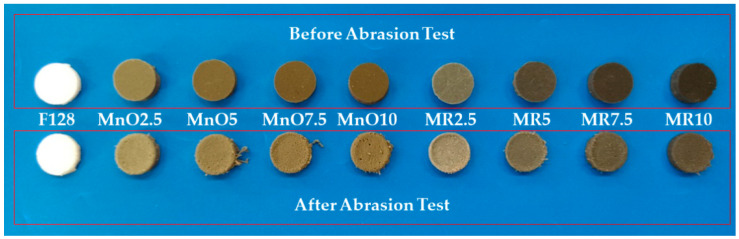
Samples before and after the abrasive wear test.

Among all of the tested materials, the highest volume loss was shown for the silicone used as a F128 matrix (0.24 cm^3^). All the composite materials demonstrated lower abrasive wear. For the material using the filler MnO 2.5, an almost two-fold reduction in volume loss was observed (0.13 cm^3^), compared to silicone F128. The MnO 5 composite is characterized by abrasion similar to that of the MnO 2.5 material, which is 0.14 cm^3^. For MnO 7.5 and MnO 10 materials, the volume loss is 0.22 and 0.21 cm^3^ respectively, which is higher than that for MnO 2.5 and MnO 5, respectively. Both values should be considered comparable. For composite materials with a higher MnO content, the volume loss is more repeatable. The lowest volume loss from composites with the manganese deposit (MR) filler, was observed for the composite with the lowest filler content—MR 2.5 (0.16 cm^3^). For the remaining composite materials, in which the filler was manganese residue, the volume loss increased with the percentage of filler content. For the MR 5 material, wear was observed to be 0.18 cm^3^, for MR material 7.5—0.19 cm^3^, and for MR 10 material—0.23 cm^3^. The volume loss of MR 10 can be considered comparable to the volume loss of pure silicone, used as matrix. The decrease in volume loss for higher filler concentrations can be explained by the fact that, at higher filler contents, they can agglomerate. During abrasion, the agglomerates of fillers may detach from the sample, which further reduces the volume of the wearing sample. Figure 13 shows photos of samples before and after the abrasive wear test process using the Schopper-Schlobach apparatus.

### 3.6. Ball-on-Disc 

**Figure 14:**
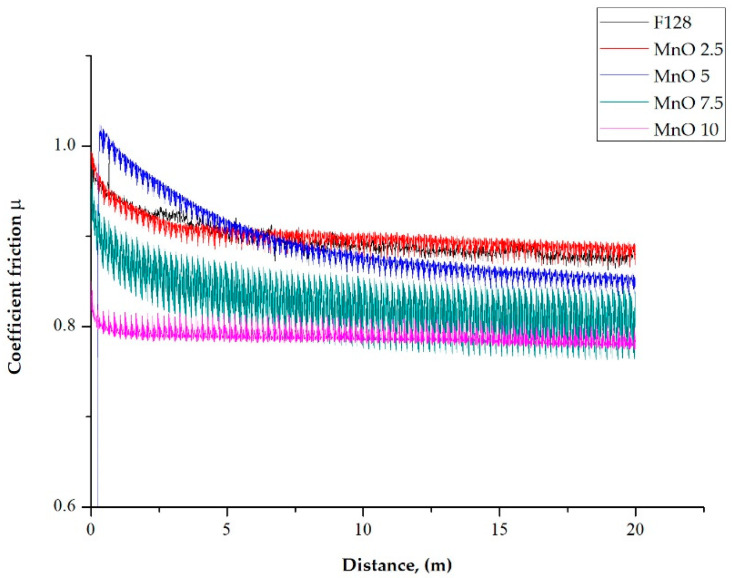
Examples of coefficient friction change in time for MnO series samples.

**Figure 15:**
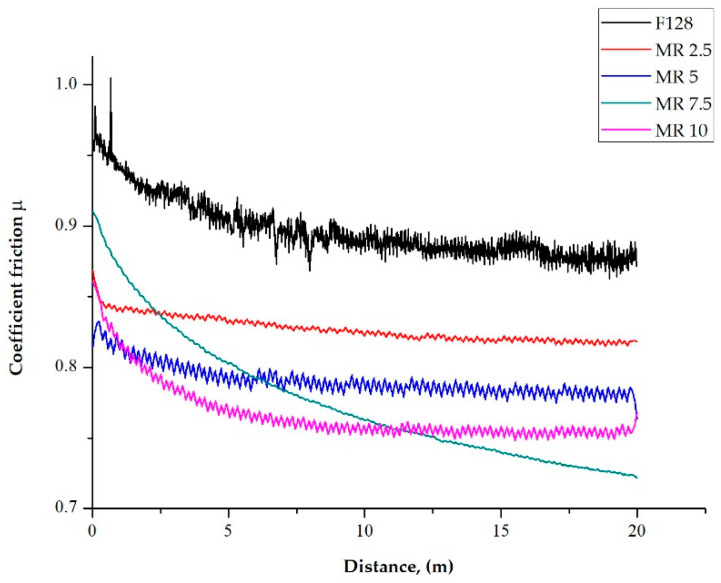
Examples of coefficient friction change in time for MR series samples.

The coefficient of friction as a function of distance for test samples, with the addition of manganese oxide (MnO series) and manganese residue (MR series), is presented as a plot in Figure 14 and Figure 15, respectively. Test was performed for three samples from each composition. Mean value of steady-state coefficient of friction from three samples from each composition is presented in Table 2. 

In all the tested samples, the value of the friction coefficient decreased in the first stage of the wear resistance test. As a result of the point contact between the test sample with a steel ball, local compressive pressure was increased, causing significant shear stress. This is equated with the Hertzian phenomenon. A similar trend was observed for both the MnO and MR test samples. Samples with a concentration of 5 wt% of MnO and 7.5 wt% of MR in the entire scope of the test did not reach the steady-state value of the friction coefficient. For the samples with a concentration of 2.5 and 5 wt% of MnO filler, reaching the steady-state coefficient friction took place after a distance d > 5 m. Increasing the concentration to 10 wt% resulted in shortening the distance to reaching the steady-state d > 1 m. While, for the samples, with a manganese residue (regardless of concentration) filler, reaching the steady-state coefficient of friction took place after a distance d > 7.5 m, which is longer as for the MnO series samples. This may suggest increased material consumption. For samples of the reference material F128, the value of the friction coefficient was characteristic for silicone viscous materials (value close to 1) and was approximately µ = 0.9 ± 0.1. Regardless of the filler used, the value of the friction coefficient (µ) of silicone material F128 decreased with an increasing concentration of fillers. In the case of the MnO series samples, increasing the filler concentration from 2.5 wt% to 5 wt% does not significantly affect the value of the friction coefficient and amounts to approximately 0.85 ± 0.07. For the MnO 10 samples, a significant decrease in the coefficient of friction values to µ = 0.78 ± 0.05 was observed, compared to the reference sample (13%). For samples with manganese residue as a filler (MR series), a concentration of 2.5 wt% resulted in a significant reduction of the friction coefficient value to µ = 0.82 ± 0.09. A further increase in the concentration of manganese residue caused a gradual decrease in the value of the friction coefficient down to µ = 0.76 ± 0.05 for MR 10 samples, which is a reduction of the friction coefficient by 16% compared to the reference material.

## 4. Discussion

Manganese residue is a waste product, where the substrate is manganese oxide. The composition of manganese oxide is declared by the manufacturer and is constant within one batch of material. The manganese residue is a waste product and its composition varies within certain limits. Depending on the raw materials used in the process and the process conditions, the chemical composition of the manganese residue may vary both qualitatively and quantitatively. A change in the chemical composition may cause changes in the values of specific physicochemical properties. Test results for composites filled with manganese oxide (MnO) and manganese residue (MR) showed similar tendencies. In the case of density, similar values of density increase were observed for MnO and MR for the same amount of filler. However, densities of compositions filled with MR filler is little higher in all analyzed cases. In terms of hardness, the materials containing the fillers showed a hardness higher than the silicone used as the matrix and showed similar hardness values for each filler content. During the tensile strength test for both MnO and MR, it was noticed that at the lowest filler concentration (2.5 wt%) the values were the lowest and the tensile strength increased with increasing filler content. It was the same with elongation at break. In the case of abrasion resistance, the highest abrasion resistance was noted for samples with the lowest content of MnO and MR, while with the increase of the filler content, the abrasion resistance decreased. However, it is worth noticing that volume loss of the silicone-based composites filled with MnO is smaller.

The introduction of these two fillers at concentrations of 2.5, 5, 7.5, and 10 wt% was carried out to reduce the abrasion of the obtained composite materials, in relation to the native silicone, when used as the matrix. In relation to the results presented in the previous publication [28], the authors found similar trends after introducing both zinc and manganese compounds into the silicone. In the case of the abrasive wear test, carried out on the Schopper-Schlobach apparatus, a decrease in the abrasive wear was noticed for all of the composites, when compared to the sample made of silicone F128. Both zinc and manganese compounds reduce the abrasive wear of silicone composites over the entire concentration range. The greatest decrease in abrasive wear is observed for composite materials in which the filler concentration is the lowest.

For silicones filled with zinc dust (ZD) at a filler concentration of 5 wt%, a decrease in abrasive wear by 58% was observed, for silicones filled with 5% sifted zinc ash (SZA) a decrease in abrasive wear by 67% was observed, and for 5 wt% zinc ash (ZA) a decrease of 63% was observed [28]. For manganese compounds, the lowest abrasive wear is observed at the lowest filler concentrations. For composites with 2.5 wt% MnO share, a 46% decrease in abrasive wear is observed, while for composites with 2.5 wt% MR share, the decrease in abrasive wear was 33%.

Both in the case of tests conducted on zinc compounds and manganese compounds, as the amount of filler increased, the abrasive wear also increased, but in the tested range, remained lower than the abrasive wear of silicone without the addition of fillers. The ball-on-disc abrasion method showed that the use of manganese oxide and manganese residue fillers had a better effect on the reduction of the friction coefficient of the silicone F128 compared to zinc metallurgical waste [28]. However, a significant change in trend of friction coefficient is not identified with respect to the wt% content of the fillers.

For composites in which zinc compounds and manganese compounds were used as a filler, it was noticed that the density of composite materials increased as the concentration of filler in the matrix increased. A similar tendency was shown for hardness. The samples filled with zinc and manganese waste were shown to have a hardness greater than the F128 silicone.

The tensile test showed that the composite materials, for all filler concentrations tested, showed worse mechanical properties than the silicone used as the matrix. Elongation at break values for composites filled with zinc compounds [28] and manganese compounds were shown to stabilize at a constant level, regardless of the filler content in the matrix.

Examples of the use of fillers introduced into a silicone matrix are described in the literature. Paper [23] shows results of the research concerning silicone filled with phosphor powders. The obtained composites were characterized by a lower tensile strength with respect to pure silicone. The paper [26] presents results of the research on composites filled with various ZrSi_2_ content. As the content of ZrSi_2_ increased, the tensile strength first decreased and then increased. The highest tensile strength was obtained for the pure silicone. Elongation at break was also the highest in case of the pure silicone. Paper [35] described research on silicone-based composites filled with organic fillers. Reported tensile strength and elongation at break of the composites in all analyzed cases are smaller than in case of the pure silicone. In our paper, tensile strength and elongation at break of the silicone-based composites filled with manganese waste in all analyzed cases are also lower than in case of the pure silicone. Paper [19] describes the results of the research on reinforced RTV composite samples filled with micro- or nano- nickel particles. Results presented in this research show that the tensile strength, elongation at break, and Young’s modulus are higher when size of the filler is lowest. Results of the research on silicone-based composites filled with Fe_3_O_4_ particles with size of <5 µm shows that the tensile strength grows with increasing amount of the filler [20]. However, elongation at break decreased with increasing amount of the filler [20]. Size of the filler grains used in our paper are larger than in case of the research described in the papers [19,20]. It is presumed that the reduction of the tensile strength would be smaller when using a filler with smaller grain size.

## 5. Conclusions

The addition of manganese-containing substances: manganese oxide and manganese residue have a positive effect on the tribological properties of the newly obtained silicone-based composites. The obtained composites in all filler concentrations (from 2.5 to 10 wt%) showed improved tribological properties and lower abrasive wear compared to the native silicone. The best properties were observed for 2.5 and 5 wt% concentration fillers added to the silicone matrix.

The obtained composites were also characterized by increased hardness, resilience, and density compared to the silicone without additives. However, the static tensile tests demonstrated a decrease in elongation at break and tensile strength compared to the native silicone. Therefore, industrial process waste, which includes manganese compounds, has a potential use for a range of fillers in silicone composites, with the potential to reduce abrasive wear and improve tribological properties. This means that there is now a useful purpose to recycling these waste products. Doing so will reduce the waste disposal process and help to take care of the natural environment. 

## Figures and Tables

**Table 1:** Composition of the samples.

Matrix	Filler	Filler Content (wt%)	Designation
Feingosil 128 PU	-	-	F128
	manganese (II) oxide	2.5	MnO 2.5
	manganese (II) oxide	5	MnO 5
	manganese (II) oxide	7.5	MnO 7.5
	manganese (II) oxide	10	MnO 10
	manganese residue	2.5	MR 2.5
	manganese residue	5	MR 5
	manganese residue	7.5	MR 7.5
	manganese residue	10	MR 10

**Table 2:** Mean value of steady-state coefficient friction.

Material	F128	MnO 2.5	MnO 5	MnO 7.5	MnO 10	MR 2.5	MR 5	MR 7.5	MR 10
Coefficient friction (µ)	0.9	0.88	0.85	0.83	0.78	0.82	0.79	0.77	0.76

## Data Availability

The data presented in this study are available on request from the corresponding author.

## Appendix A

**Table A1:** Properties of silicone Feingosil 128 PU [29].

Properties	Unit	Value
Ratio mixing A:B (silicone Feingosil 128 PU: catalyst PES)	-	100:5
Density	g/cm^3^	1.2
Viscosity	mPas	30000
Hardness	ShA	20
Linear shrinkage	%	<0.7
Elongation at break	%	200–300
